# Cloning and characterization of *EgGDSL*, a gene associated with oil content in oil palm

**DOI:** 10.1038/s41598-018-29492-6

**Published:** 2018-07-30

**Authors:** Yingjun Zhang, Bin Bai, May Lee, Yuzer Alfiko, Antonius Suwanto, Gen Hua Yue

**Affiliations:** 1 Temasek Life Sciences Laboratory, 1 Research Link, National University of Singapore, 117604 Singapore, Singapore; 2 Institute of Cereal and Oil Crops, Hebei Academy of Agriculture and Forestry Sciences, 162 Hengshan Street, Shijiazhuang, 050035 China; 3 Wheat Research Institute, Gansu Academy of Agricultural Sciences, 1 Nongkeyuanxincun, Lanzhou, 730070 China; 4 Biotech Lab, Wilmar International, Jakarta, Indonesia; 5 Bogor Agricultural University, Bogor, Indonesia; 6 Department of Biological Sciences, National University of Singapore, Singapore, Singapore; 7 School of Biological Sciences, Nanyang Technological University, 6 Nanyang Drive, 637551 Singapore, Singapore

## Abstract

Oil palm (*Elaeis guineensis*, Jacq.) is a key tropical oil crop, which provides over one third of the global vegetable oil production, but few genes related to oil yield have been characterized. In this study, a GDSL esterase/lipase gene, which was significantly associated with oil content, was isolated from oil palm and designated as *EgGDSL*. Its functional characterization was carried out through ectopic expression in *Arabidopsis* ecotype Col-0. It was shown that expression of *EgGDSL* in *Arabidopsis* led to the increased total fatty acid content by 9.5% compared with the wild type. Further analysis of the fatty acid composition revealed that stearic acid (18:0) increased in the seeds of the transgenic lines, but the levels of linoleic acid (18:2) plus 11-eicosenoic acid drastically declined. Quantitative real-time PCR (qPCR) revealed that in oil palm, *EgGDSL* was highly expressed in mesocarp followed by leaf, and the expression level was very low in the root. The expression level of *EgGDSL* gene began to increase at two months after flowering (MAF) and reached its peak by four MAF, then declined rapidly, and reached its lowest level during the mature period (6 MAF). The *EgGDSL* gene was more highly expressed in oil palm trees with high oil content than that with low oil content, demonstrating that the transcription level of *EgGDSL* correlated with the amount of oil accumulation. The gene may be valuable for engineering fatty acid metabolism in crop improvement programmes and for marker-assisted breeding.

## Introduction

Oil palm (*Elaeis guineensis*, Jacq.) is an important tropical oil crop mainly distributed in the belt between 15 degrees north latitude and 10 degrees south latitude. The mesocarp and endosperm of oil palm are well known for their high oil content, which produces two major economic important oils, palm oil and palm kernel oil, respectively^1^. Palm oil is a very versatile oil because its uses are not limited to food only but also widely used in non-food applications such as in cosmetics, pharmaceuticals, lubricants and can even serve as biofuel^1,2^. Oil palm is a kind of crop with very high economic value. For each hectare of oil palm, which can be harvested year-round, the annual production averages 20 tons of fruit yielding 4,000 kg of palm oil and 300 kg of palm kernel oil. Furthermore, as a perennial crop, it can be harvested for 25–30 years once planted^2^.

Vegetable oil production and consumption increased rapidly in recent years. Palm oil, accounting for over 1/3 of total edible oil production, increased significantly at an annual rate of 6% in the past decade^1^. Traditional breeding has played an important role in the increase of palm oil production. By using *Tenera* genotype derived from the cross between *Dura* and *Pisifera*, the yield of palm oil increased from 2.0 to 4.1 tons ha^−1^ in the last 50 years. However it was still much lower than the estimated theoretical maximum yield of more than 18.2 t ha^−1^ ^1^. Yield improvement through traditional breeding technique has encountered a bottleneck because of the narrow gene pool and the long generation cycle of oil palm (10–20 years)^2^. Therefore, an effective way to continue to increase oil production is to find oil biosynthesis related genes and use them for elite oil palm germplasms breeding via gene pyramiding or marker-assisted selection^3^. Genetic engineering will be an especially good way for modifying oil palm as it can greatly reduce the cost and time for improving the oil yield trait of oil palm^3^. Research has shown that this is a viable approach, because good results have been achieved to improve the quality or quantity of seed storage oils via the genetic engineering of lipid metabolic pathways in maize^4^, soybean^5^, oilseed rape^6^ and other oil crops^7–9^.

Oil biosynthesis processes have been shown to be far more complex than a simple linear pathway^10^, and are controlled by multigenes, which makes studies difficult. To date, several genes in this pathway have been identified, such as *PDAT*^11^, *DGAT*^12,13^, *WRI1*^14^. But, few genes have been functionally characterized in oil palm. Further research is required to find oil biosynthesis related genes that can be used to improve oil content in oil palm. Most studies in oil palm focused on QTL mapping for yield related traits. For example, QTL analyses have been conducted for the traits of bunch number, fresh fruit bunch yield, oil yield, oil to bunch content and fatty acid composition^15–20^ and leaf area^21^. With the advent of next-generation sequencing (NGS) and genotyping by sequencing (GBS), genome-wide association studies (GWAS) have been used to identify the oil yield-related loci^22–24^. A GWAS for oil to dry mesocarp content (O/DM) on 2,045 *Tenera* palms was performed using 200 K SNPs and identified 80 loci which were significantly associated with O/DM^23^. However, none of the genes located in these identified loci have been characterized. The whole genome sequences of oil palm have been published^2,25^, which provided us a powerful reference to find the candidate genes conferring with the oil yield trait.

The aims of this study were based on the GWAS results of Teh *et al*.^23^ (a) to identify and characterize candidate genes responsible for variation in oil content, (b) to analyze the effect of candidate genes on oil content and fatty acid composition by ectopic expression in *Arabidopsis*, (c) to elucidate the gene expression pattern of candidate gene in oil palm. Our results could provide important information for improving oil yield of oil palm by genetic engineering approach.

## Materials and Methods

### Palm materials

Leaves, roots and developing fruits of oil palm were used to isolate RNA for the analysis of gene expression patterns. All plant materials were derived from a cross between *Deli Dura* and *Ghana Pisifera* from the Wilmar International Plantation. The trees were planted in the same field in 2006 and managed under the same conditions in Indonesia. The field arrangement was carried out following the standard protocol of Wilmar International Plantation. The oil to bunch content (O/B) was recorded over three periods from 2012 to 2014 and the oil to dry mesocarp content (O/DM) was recorded for two years from 2012 to 2013. Oil palm fruits from different developmental stages were collected from the same tree to analyze *EgGDSL* gene expression changes. Flowers were tagged at the anthesis period and fruits were collected at 2, 3, 4, 5 and 6 months after flowering (MAF). The fruits of 4 MAF from six trees with different O/DM contents were used to check the *EgGDSL* gene expression level. The trees were T01 (84.34%, O/DM), T02 (82.67%), T03 (78.11%) T04 (76.37%), T05 (76.17%) and T06 (72.94%). They had stable O/DM and O/B content in different years (Table S1). All the samples were collected with three biological repeats.

### Selection of candidate genes associated with oil content

Candidate genes were selected based on the GWAS data published^23^. The GWAS for oil-to-dry-mesocarp content on 2,045 *Tenera* palms was performed using 200 K SNPs and found that 80 loci were associated with O/DM. Most loci were clustered on chromosome 5. Therefore, we mainly focused on the significant SNPs on chromosome 5. By setting a significance cutoff (*p*) at 10^−5^, we selected 42 significant SNPs (Table S2) to do further analysis. Then each SNP was anchored onto the oil palm physical map by BLAST searching against the oil palm genome database using the flanking sequence of SNP as a probe. The significant SNPs were mainly in scaffold 3 and scaffold 94. A sketch of annotated genes and significant SNPs in scaffold 3 was made to find the candidate genes (Fig. S1). Based on the distance with significant SNPs and annotated function, two genes seemed to have high probabilities of involvement in the oil biosynthesis pathway. One was p5_sc00003.V1.gene1598 which was annotated as GDSL esterase/lipase and there were four significant SNPs (SNP47116, SNP46882, SNP47117 and SNP47120) within a distance of 18 kb; this gene was abbreviated to *GDSL*. The other was p5_sc00003.V1.gene1547 with annotated function as pentatricopeptide repeat protein, on the account that many esterase/lipase sequences possess the pentapeptide motif and there were three significant SNPs (SNP22773, SNP22774 and SNP19028) in this short distance; this gene was called *PPR* (pentatricopeptide repeat protein) for short.

### Phylogenetic analysis and predicting the protein structure of selected candidate gene EgGDSL

A total of 90 genes annotated as encoding GDSL esterase/lipase in oil palm were identified from GenBank. As *EgGDSL* in this research was annotated as GDSL esterase/lipase *At1g71250*-like gene, the 10 homologous genes having the highest similarity with *At1g71250* in *Arabidopsis* were selected as well. The deduced protein sequences were aligned using multiple sequence alignments via ClustalW method. Nineteen GDSL genes in oil palm were excluded from the alignment because of the poorly matched alignable regions. The remaining sequences consisting of 71 *EgGDSL* and 10 *AtGDSL* genes were used to do phylogenetic analysis (Table S3). The unrooted phylogenetic tree was constructed using the UPGMA method and was displayed using the MEGA 4 software^26^. The bootstrap values of 1,000 replicates were placed at the nodes. The Multiple Em for Motif Elicitation motif search tool (MEME, http://meme-suite.org/tools/meme) was employed to identify the putative conserved motifs in the GDSL family. We analyzed the characteristics of the deduced EgGDSL protein using SOSUI analysis (http://harrier.nagahama-i-bio.ac.jp/sosui/sosui_submit.html) and ProDom (http://prodom.prabi.fr/prodom/current/html/home.php). Furthermore, the secondary and tertiary structures of the EgGDSL protein were determined by I-TASSER (http://zhanglab.ccmb.med.umich.edu/I-TASSER).

### Construction of gene expression vectors for candidate genes and analysis of expression in Arabidopsis

Genomic DNA was extracted from leaf samples of oil palm germplasms DuraB and TS3 (DuraB was a *Deli Dura* tree and TS3 was a *Ghana Pisifera* tree). The primers GDSL-FL and PPR-FL (Table 1) were designed using the software Premier Primer V5.0 (http://www.premierbiosoft.com) to amplify the full-length sequences of genes *GDSL* and *PPR*, respectively. The italic characters in forward primers indicate the restriction enzyme *Asc*I recognition site and that in reverse primers were *Spe*I recognition site. In order to conduct western blot analysis, a FLAG tag sequence (characters with underline in forward primers) was added before the start codon. PCR was performed in total volumes of 20 µl, including 2 µl of 10× Pfu buffer, 0.5 µl of dNTP (2.5 mM of each dNTP), 1 µl of each primer (3 µM), 1 U of Pfu DNA polymerase and 20 ng of template DNA. Reaction conditions were 94 °C for 5 min, followed by 35 cycles of 94 °C for 45 s, annealing at 54 °C for 45 s, 72 °C for 2 min, and a final extension of 72 °C for 5 min. PCR product was separated by electrophoresis on 1% agarose gel, the desired band was recovered and introduced into the pGEM-T easy vector (http://www.promega.com) for sequencing. The sequences of both *GDSL* and *PPR* genes of oil palm were submitted into GenBank database.

**Table 1:** The primer sets used in this study.

Primer name	Sequence (5′-3′)	Amplified target
GDSL-FL	Forward: TT*GGCGCGCC*ATGGATTACAAGGATGACGACGATAAGATGGGACGAAAGCTTCTTCTTCTT	Full-length sequence of gene *EgGDSL*
	Reverse: GG*ACTAGT*TCACAGCCATCCATCAGATGCA	
PPR-FL	Forward: TT*GGCGCGCC*ATGGATTACAAGGATGACGACGATAAGATGCCATATCTTCACGGGAGC	Full-length sequence of gene *EgPPR*
	Reverse: GG*ACTAG*TTCACCAAAAGTCATTGCAAGAACA	
Actin	Forward: GAGAGAGCGTGCTACTCATCTT	qPCR primers for β-*actin* gene
	Reverse: CGGAAGTGCTTCTGAGATCC	
GAPDH	Forward: GATCGAGAAATCAGCCACGTATG Reverse: GTCACCAATAAAGTCGGTGGACA	qPCR primers for glyceraldehyde 3-phosphate dehydrogenase gene
qPCR-GDSL	Forward: CGCCCTCTTCATCCTCTCCT	qPCR primers for gene *EgGDSL*
	Reverse: TTACCCTGATGGCCTGGATG	

The binary vector pBA002 was used to perform gene expression in *Arabidopsis*. The vector pBA002^27^ was provided by Professor Nam Hai Chua’s Lab from Temasek Life Sciences Laboratory, National University of Singapore, Singapore. The exogenous gene was driven by the 35S CaMV promoter and the herbicide-resistant *Bar* gene was used as a selectable marker for transformation positive seedling screening. The positive alleles of *GDSL* and *PPR* gene in TS3 were introduced into pBA002 by *Asc*I/*Spe*I double digestion and ligation, respectively, because *Pisifera* normally has a higher O/DM content than *Dura*. Finally, the recombinant vector was transferred into *Agrobacterium* strain GV3101, which was used to transform *Arabidopsis*.

### Growing Abrabidopsis and transformation using floral dip method

Seeds of *Arabidopsis* ecotype Columbia (Col-0) were sown onto planting soil (3:1 mixture of peat moss: sand) in a 32-cell plant seedling tray and grew in a controlled growth chamber (22 °C and 16 h photoperiod). Nine seedlings were planted in one cell and watered once a week. Agrobacterium-mediated transformation was performed when the seedlings were in blossom period using to the floral dip method reported^28^. After all siliques were formed, watering was reduced to dry the seeds. Mature seeds were harvested and dried for 10 days to break seed dormancy. The transgenic positive seedlings were planted under the same conditions but only one seedling per cell.

### Screening primary transformants

Primary transformants can be directly screened during growth by spraying with the herbicide glufosinate ammonium as the binary vector PBA002 carries the *bar* gene selection marker. T_1_ generation seeds were sown onto moist soil and herbicide spraying was initiated when the cotyledons were visible. We sprayed the seedlings twice a week with a glufosinate ammonium solution (10 mg L^−1^). Transformants continued developing, while non-transformed seedlings turned yellow after 2–3 weeks. The transformants were transplanted to a new plant seedling tray with a single seedling per cell. According to the ratio of offspring resistant/susceptible to glufosinate ammonium, seedlings with the single-copy gene transformation in the T3 generation were selected and used for further analysis.

### Western blot analysis to screen transformants

We performed western blot analysis to further screen the transformants. 30 mg of leaf sample of each primary transformant was taken for western blot analysis using the anti-FLAG antibody according to the method reported by Einhauer and Jungbauer^29^. Total proteins were isolated using 50 µl of extraction buffer (Bio-rad, Laemmli Sample buffer) supplemented with 5% β-Mercaptoethanol and separated by 8% SDS-PAGE gel. Proteins were transferred to PVDF membrane overnight at 4 °C and non-specific binding sites were blocked by immersing the membrane in 3% non-fat dried milk. The membrane then was incubated in the incubation buffer with the diluted primary antibody (Sigma, Monoclonal ANTI-FLAG M2 antibody produced in mouse) and secondary antibody (Amersham, ECL anti-mouse lgG, horseradish, peroxidase linked whole antibody from sheep). After washing the membrane, the mixed detection reagent (Bio-rad, Clarity Western ECL Substrate) was added onto the membrane. Finally, the membrane was scanned using the ChemiDoc Touch Imaging System (Bio-Rad) and the results were recorded.

### Determining fatty acid content in seeds of transgenic lines

In order to find out whether the selected candidate genes are associated with oil biosynthesis, we analyzed total fatty acid content of transformants. The triacylglycerols (TAGs) are the major constituent of vegetable oil, which consist of glycerol esterified with three fatty acids^30^, Therefore, the total fatty acid content can basically explain the oil content. Mature seeds of transformants were harvested and cleaned to remove as debris. Fatty acid content assays were conducted according to the method reported by Li^31^ with minor changes. Dried seeds (about 20 mg) were weighed on an analytical balance and then put into a glass tube with PTFE-lined screw-cap. One ml of 5% (v/v) conc. sulfuric acid in MeOH (freshly prepared for each use), 25 µl of BHT solution (0.2% butylated hydroxyl toluene in MeOH), 150 µg of triheptadecanoin (as a triacylglycerol internal standard to generate methyl heptadecanoate) and 300 µl of toluene as co-solvent were added to the tube. The mixture was heated at 93 °C for 2 h. After cooling to room temperature, 1.5 ml of 0.9% NaCl (w/v) and 2 ml hexane were added. The mixture was vortexed for 15 min and centrifuged at 4,500 rpm for 10 min. 500 µl of supernatant was taken to perform total fatty acid content analysis. The fatty acid methyl esters (FAMEs) extracts were analyzed with GCMS (QP2010, Shimadzu, Japan) equipped with an HP-88 fused silica capillary column (30 m length, 0.25 µm diameter and 0.25 mm film thickness, Agilent J & W Scientific, Folsom). The running conditions were: split mode injection (1:10), injector temperature at 250 °C and the oven temperature program (starting at 70 °C for 1 min, increasing to 200 °C at the rate of 25 °C/min and holding for 1 min, then increasing to 230 °C at 5 °C/min and holding for 1 min, finally increasing to 250 °C at 50 °C/min and holding for 2 min). The FAME peaks were identified by searching against the Shimadzu NIST08 compound database. The area of peaks in the GC chromatogram was used to calculate the mass of total fatty acid in the sample by comparison to the internal standards. The percentage of each fatty acid was calculated by area percentage method.

### Measuring seed weight and seed size

Mature *Arabidopsis* seeds (500 seeds per replicate, three replicates) were counted and oven dried at 37 °C for 24 h to ensure that all the seed samples analyzed contain equal water content. The samples were immediately weighed carefully on an analytical balance. Thirty seeds were put on the stage of a dissecting microscope and a picture was taken using a camera. The values of seed length and seed width were directly obtained from the images by employing the image analysis software Image-Pro Plus 6.0 (http://www.mediacy.com).

### Real-time quantitative PCR for EgGDSL gene expression pattern in oil palm

Total RNA from leaves, roots and fruits of oil palm were isolated using RNeasy Plant Mini Kit (Qiagen) and digested with DNase I to remove the genomic DNA. First-strand cDNA was synthesized from 2 µg of total RNA using the MMLV reverse transcriptase (Promega). All qPCR primers (Table 1) were designed by using Primer-Blast (https://www.ncbi.nlm.nih.gov/tools/primer-blast). Real-time PCR analysis was performed using CFX96 Real-Time System (Bio-Rad). SYBR Green I was used as the dye for detection. *β*-*actin* gene and glyceraldehyde 3-phosphate dehydrogenase gene (*GAPDH*) were used for normalization. PCR efficiency was established by means of calibration curves using diluted cDNA with a 5 times concentration gradient. The qPCR was programmed as an initial denaturation at 95 °C for 3 min, followed by 95 °C for 10 s and 60°C for 30 s, 40 cycles. Expression level was quantified in terms of comparative threshold cycle (C_T_) using Vandesompele method^32^. The experiments were performed in triplicate for each gene.

### Statistical analysis

Boxplot was employed to assess the total fatty acid content differences between transformants and wild-type *Arabidopsis*. Data were analyzed with ANOVA and Student’s t-test using Statistical Analysis System (SAS Institute, V9.3).

## Results

### Cloning and characterization of genes EgGDSL and EgPPR in oil palm

Based on the GWAS data published^23^, we found that the gene *GDSL* and *PPR* could be the candidate genes associated with the difference of oil content traits. By PCR we obtained the full-length sequences of *EgGDSL* and *EgPPR* of Dura B and TS3 (*Pisifera*), respectively. The genome sequence of *EgGDSL* was 840 bp and contained two exons. The coding sequence and deduced polypeptides of *EgGDSL* were 735 bp and 244 amino acids, respectively. There were seven single nucleotide polymorphisms (SNPs) between Dura B and TS3 with 1 SNP in the intron and 6 SNPs in the second exon region; the two alleles were designated as *EgGDSL-5D* (GenBank accession KY911277) and *EgGDSL-5P* (GenBank accession KY911278), respectively (Fig. S2). Both *EgGDSL-5D* and *EgGDSL-5P* contained complete open reading frames (ORF), but the 6 SNPs in exon 2 caused 4 deduced amino acids differences between DuraB and TS3 (Fig. S3). The genome sequences of *EgPPR* of DuraB and TS3 were 2,498 bp and 2,494 bp, respectively. Gene *EgPPR* contained 2 exons and the coding sequence was 1,830 bp. There was a 4 bp indel in the intron region and 4 SNPs in the exon region between DuraB and TS3; the two alleles were designated as *EgPPR-5D* (GenBank accession KY944570) and *EgPPR-5P* (GenBank accession KY944571), respectively (Fig. S4). The SNPs caused 4 deduced amino acids differences between DuraB and TS3 (Fig. S5).

### Phylogenetic and protein motifs analysis of GDSL

**Figure 1:**
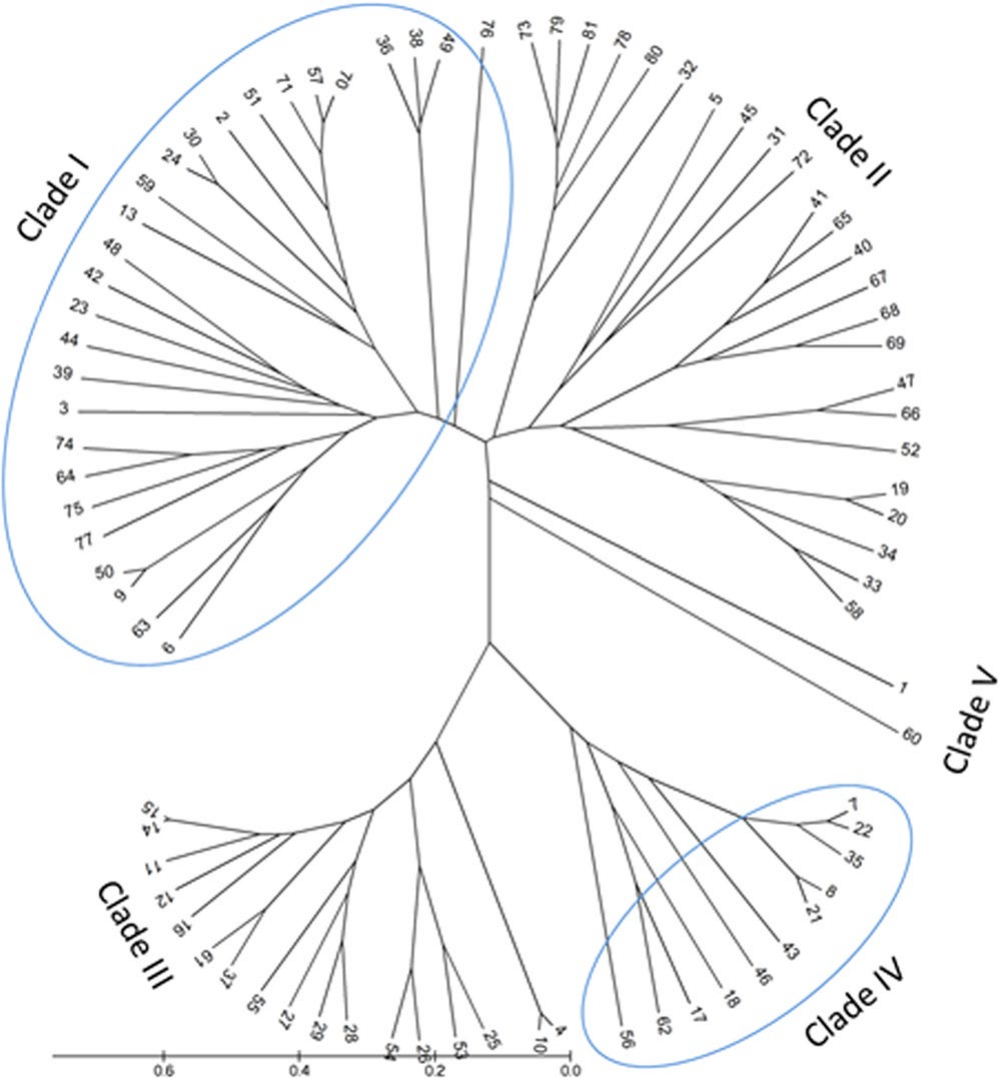
The phylogenetic relationship of the GDSL family. The unrooted tree was constructed based on multiple sequence alignment of the deduced GDSL protein sequences using ClustalW program by UPGMA method with 1,000 bootstrap replicates. Clades are numbered and the scale bar corresponds to 0.2 estimated amino acids substitutions per site. Details of the gene names (1–81) can be found in Supplementary Table S3.

To construct a phylogenetic tree to understand the relationships among *GDSL* genes, a dataset of 81 protein sequences from the GDSL family, comprised of 71 from oil palm and 10 from *Arabidopsis*, were collected (Table S3). These were divided into five clades in the unrooted phylogenetic tree, and each clade could be further subdivided into several subclades (Fig. 1). According to the results, the *EgGDSL* gene (gene number 1, KY911278) was very different from other genes except one gene (XM_010931430); these two genes were grouped into one clade (clade V). The 10 *GDSL* homologous genes of *Arabidopsis* were grouped into clades I and II. Among these, three genes *AT1G71250*, *AT5G08460* and *AT5G15720* had high similarity and were grouped into a subclade. Another five genes, *AT5G40990*, *AT3G14225*, *AT1G53940*, *AT1G53920* and *AT1G53990*, were grouped into another subclade. Though *EgGDSL* was annotated as GDSL esterase/lipase *At1g71250*-like gene, they were distant in relationship and divided into different clades.

**Figure 2:**
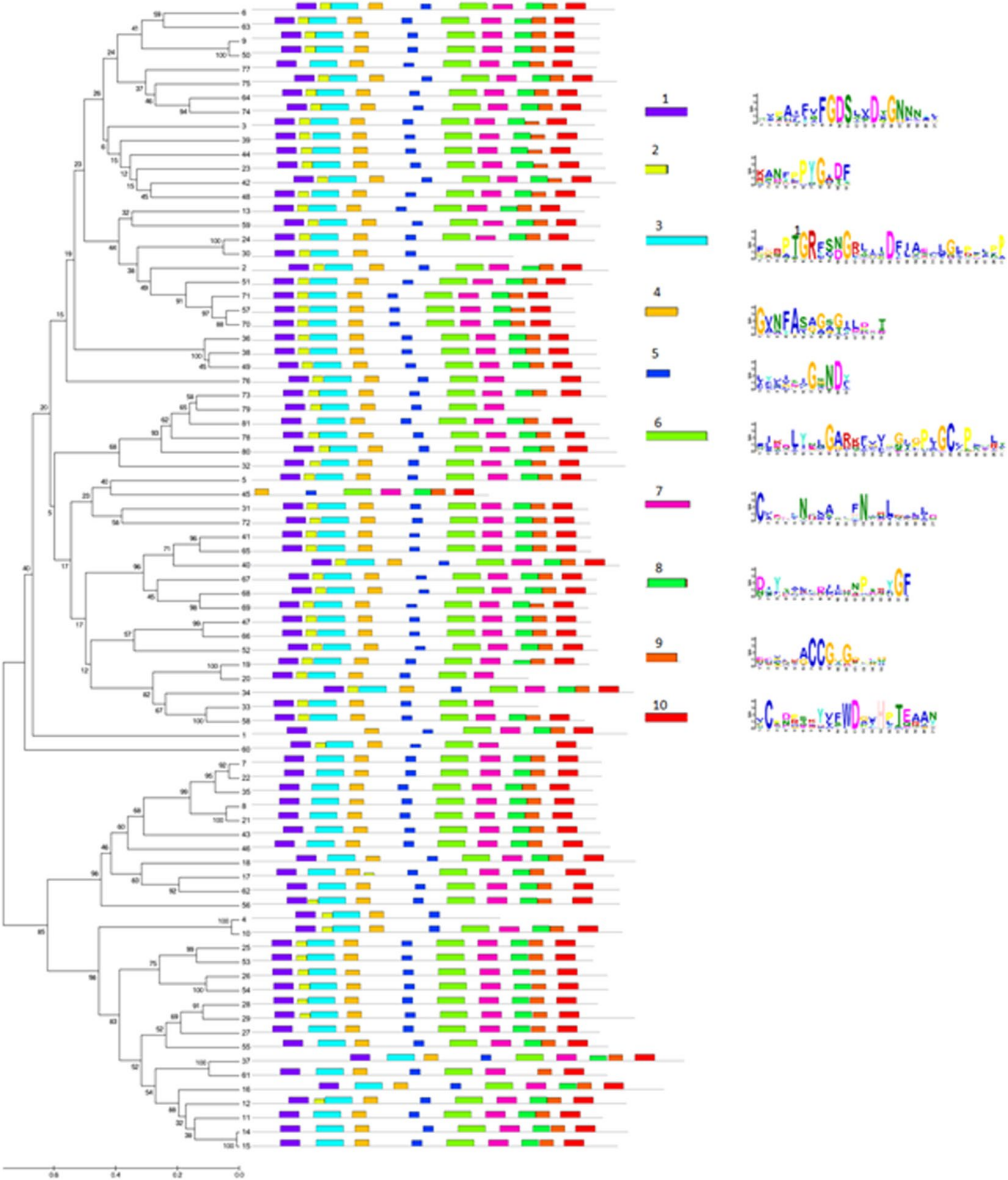
Analytical view of protein motifs view with phylogenetic analysis of GDSL esterase/lipase. Protein motifs among GDSL esterase/lipase were found using MEME software and the top 10 motifs with statistical significance (E-value) from 3.8e-251 to 2.6e-881 were selected; they were in different colours. Each motif sequence was showed on right part of the figure.

Protein motifs among GDSL esterase/lipase were found using MEME software. The top 10 motifs with statistical significance (E-value) from 3.8e-251 to 1.3e-1053 are shown in Fig. 2. Most of the GDSL proteins were detected with the ten conserved motifs. Motifs 1 (2.2e-717), 2 (3.8e-251), 5 (7.4e-254) and 10 (2.6e-881) represent GDSL esterase/lipase conserved blocks I, II, III and V, respectively. The EgGDSL protein had 8 motifs. It lacked motifs 2 and 3, which meant that block II was absent in EgGDSL esterase/lipase. In *Arabidopsis*, *AT5G15720* and *AT1G53990* lacked motif 2, *AT2G039800* lacked motifs 8 and 9, and *AT1G53940* lacked 8, 9 and 10. *AT1G71250*, the gene most homologous to *EgGDSL*, had all the 10 motifs.

### The protein structure of selected candidate gene EgGDSL

We analyzed the characteristics of the deduced EgGDSL protein. SOSUI analysis suggested that EgGDSL was a membrane protein which had one transmembrane helix. ProDom identified the presence of certain domains and showed that EgGDSL mainly contained two conserved protein domains: PDB14563 and PDD020T4 which were in amino acid region 42–125 and 186–225, respectively. Both the two domains belonged to lipase/acylhydrolase family. There were six SNPs in the second exon of *EgGDSL* gene between DuraB and TS3, which caused four amino acid substitutions. The first two amino acid substitutions were in the PDB14563 domain. Furthermore, the secondary and tertiary structures of the EgGDSL protein were determined by I-TASSER. There were three amino acid substitutions (the 93^th^, 163^th^ and 170^th^ amino acids) in the β-strand, one of the secondary protein structures. There were also some differences in tertiary protein structures between EgGDSL-5D and EgGDSL-5P (Fig. S6).

### Expression of the EgGDSL gene in Arabidopsis led to increased fatty acid content

**Figure 3:**
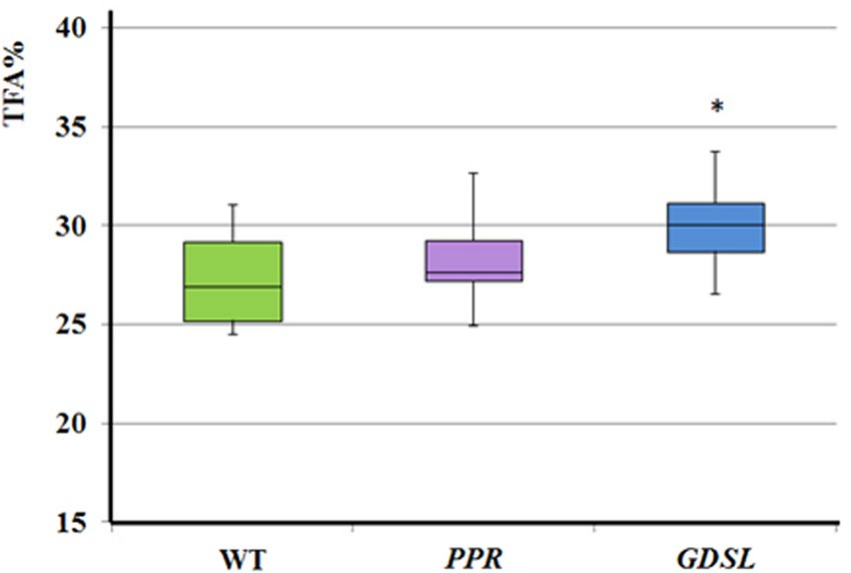
Total fatty acids content (TFA%) of wild-type *Arabidopsis* and transgenic lines. The boxplot represents median values, percentile 25–75 and outliers for the total fatty acids content of the wild-type and transgenic lines. Statistical significance was determined by a Student’s t-test at *P* < 0.01.

The full-length genome sequences of *EgGDSL* (KY911278) and *EgPPR* (KY944571) were introduced into *Arabidopsis* plants. In total, 18 wild-type Col-0 seedlings were transformed for each gene by floral dip assay. A total of 18 *EgGDSL* transformation lines were detected and 15 lines had proper protein binding signal; the positive rate was 83.3%. 9 *EgPPR* transgenic lines had correct protein binding signal, with a positive rate of 21.9% (Fig. S7). In the T3 generation, 24 transgenic lines with single-copy insertion of *EgGDSL* and *EgPPR* genes were collected separately and each independent transgenic line was analyzed for total fatty acid (TFA) content. The TFA were determined via direct methylation and GCMS in three sets of parallel experiments. The TFA content in *Arabidopsis* wild type was found to range from 24.48–31.03% and the median value was 26.97% which fell in the range reported in the literatures for Col-0 wild type seeds. The transgenic lines of *EgGDSL* gene had much higher TFA than wild-type; the range was 26.54% to 33.75% and the median was 29.80%. However, the TFA content in *EgPPR* transgenic lines (median was 27.65%) were similar to the wild-type (Fig. 3). We also performed Student’s *t*-test at *P* < 0.01 to analyze the significance of differences between wild-type and transgenic lines. The results showed that the mean TFA of *EgGDSL* transgenic populations was significantly higher than the wild-type. While wild-type seeds were observed to contain 27.31% of TFA on average, *EgGDSL* transgenic lines presented 29.91%. *EgGDSL* transgenic lines increased by 9.5% compared with non-transgenic seeds. In contrast, no significant difference of TFA was obtained between *EgPPR* transgenic lines and the wild-type, suggesting that the impact of *EgPPR* gene on oil biosynthesis was small.

### The effect of EgGDSL gene on fatty acid composition

**Figure 4:**
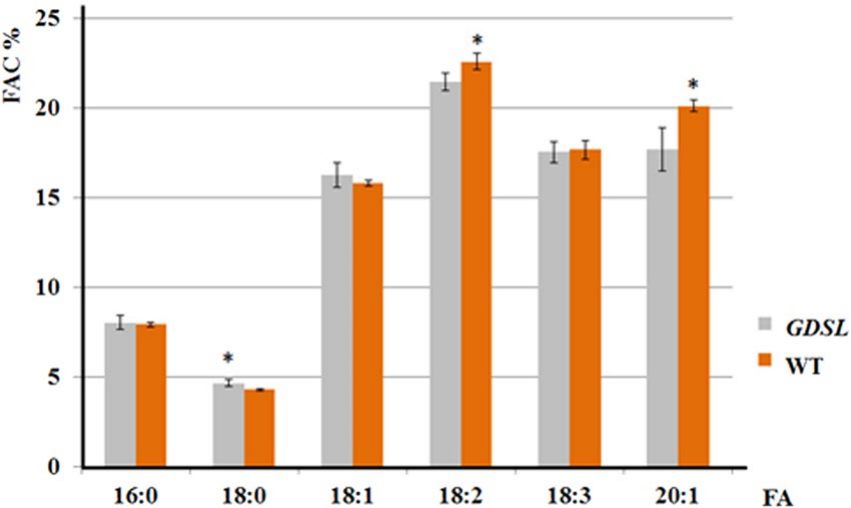
Fatty acids composition (FAC%) of wild-type *Arabidopsis* seeds and *EgGDSL* transgenic lines. The X-axis shows the names of the fatty acids, where the first number corresponds to the number of carbon atoms in the chain and the second is the number of double bonds. Error bars depict standard deviation (±SD) and asterisks indicate a significant difference between the wild-type and transgenic lines (*P* < 0.01) determined using Student’s t-test.

A total of 13 fatty acids were examined for each sample using gas chromatography-mass spectrometry (GCMS). The six principal fatty acids were palmitic acid (16:0), stearic acid (18:0), oleic acid (18:1), linoleic acid (18:2), linolenic acid (18:3) and 11-eicosenoic acid (20:1). Analysis of fatty acid composition (FAC) revealed that *EgGDSL* transgenic lines had some differences (*P* < 0.01) in profile compared with wild-type (Fig. 4). The stearic acid (18:0) increased significantly in *EgGDSL* transgenic lines on a percentage basis, whereas the levels of linoleic acid (18:2) and 11-eicosenoic acid were decreased drastically. The palmitic acid (16:0), oleic acid (18:1) and linolenic acid (18:3) did not change significantly.

### EgGDSL transgenic lines with high fatty acid content had heavier seed weight and larger seed size

**Figure 5:**
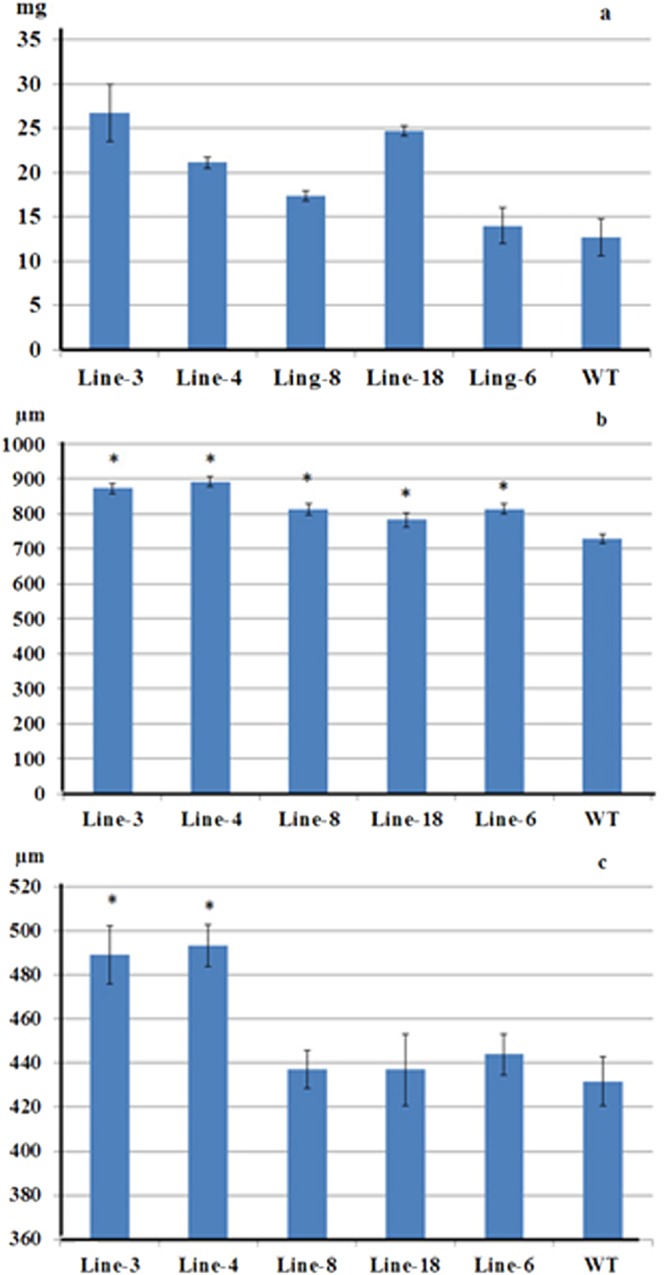
Comparisons of seed weight, seed length and seed width between transgenic lines and wild-type *Arabidopsis*. (**a**) Thousand seed weight; (**b**) seed length; (**c**) seed width. The values present mean ± SD and asterisks indicate significant difference at *P* < 0.01.

To examine whether the expression of *EgGDSL* gene affected the morphological changes in transgenic *Arabidopsis* seeds, the thousand-seed weight, seed length and seed width of transgenic lines and wild-type were measured. We chose the top 5 transgenic lines with high fatty acid content for analysis; they were line-3 (32.56%, TFA%), line-4 (31.17%), line-6 (31.25%), line-8 (31.85%) and line-18 (29.88%). The three transgenic lines (line-3, line-4 and line-18) had significantly higher thousand-seed weights than the wild-type (12.7 mg). The seed weights of these three transgenic lines (i.e. lines-3, -4 and -18) increased by 66.1–110.2% compared to wild-type. On the other hand, line-8 and line-6 were only slightly heavier than the wild type (Fig. 5a). The seed length and seed width were inspected under a microscope as well, and the values represented mean ± SD of thirty seeds (Fig. 5b,c). The seed lengths of all the 5 transgenic lines were significantly larger than the wild-type (*P* < 0.01). The seed widths of line-3 and line-4 increased significantly compared with non-transgenic seeds (*P* < 0.01), whereas that of the other 3 lines (line-8, -18 and -6) were similar to wild-type. These results demonstrated that high oil content normally led to heavier seed weight and larger seed size, especially in seed length.

### EgGDSL expression pattern in oil palm

**Figure 6:**
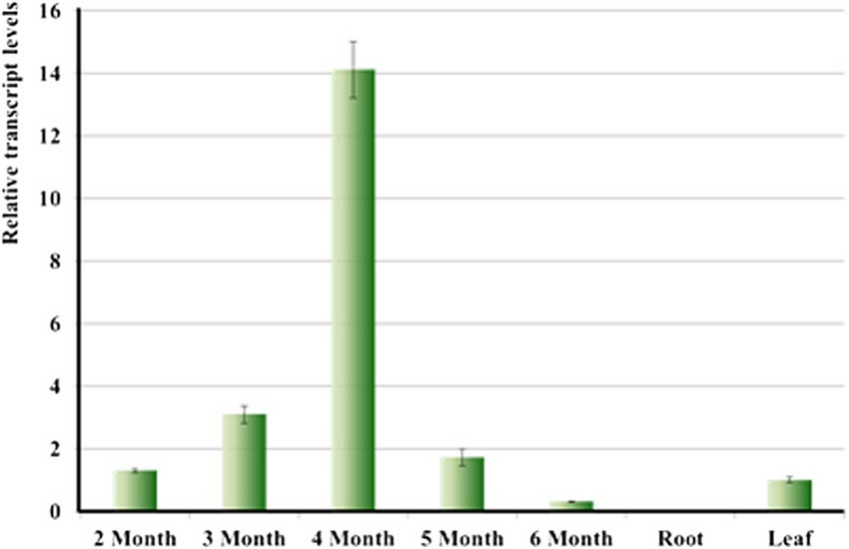
Relative transcript levels of *EgGDSL* gene in mesocarp (2 months to 6 months after flowering), root and leaf tissues. Error bars represent standard errors of the mean (±SEM).

**Figure 7:**
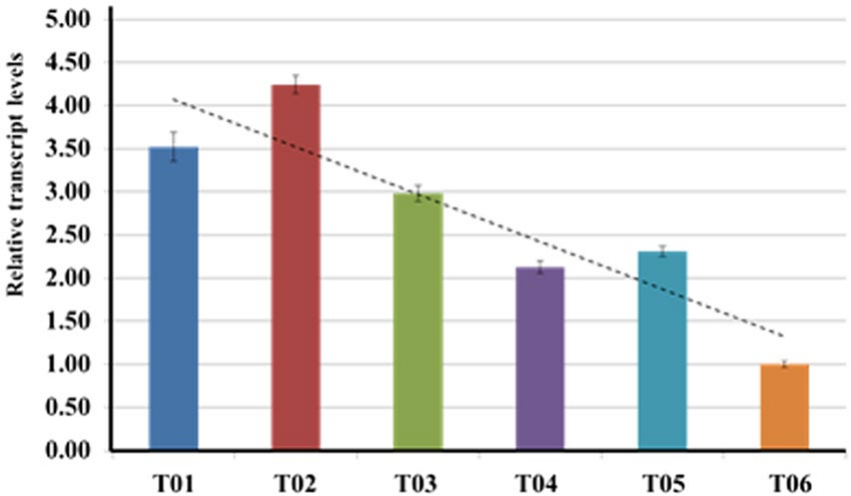
Quantitative analysis of *EgGDSL* transcript level in oil palm trees with different oil content. Transcript levels were measured by qPCR. Values represent mean ± SEM of three independent experiments. The dotted line means general trend of *EgGDSL* expression with O/DM.

In order to check the expression pattern of *EgGDSL* in oil palm, quantitative real-time PCR (qPCR) was performed to examine the transcription levels of *EgGDSL* gene in leaf, root and mesocarp. All qPCR primer sets had good amplification efficiency: 100.9 (*R*^2^ = 0.998) for β-*actin*, 101.0 (*R*^2^ = 0.999) for *GAPDH* and 99.5 (*R*^2^ = 0.998) for *EgGDSL*. *EgGDSL* was highly expressed in mesocarp followed by leaf, and the expression level was very low in root. *EgGDSL* gene expression level changes with different developmental stages of the fruit (Fig. 6). It began to increase at 2 MAF and reached its peak by 4 MAF, then declined rapidly, and reached its lowest level during the mature period (6 MAF). The *EgGDSL* expression level at 4 MAF was tens of times higher than that at 6 MAF (Fig. 6). The result indicated that *EgGDSL* was highly expressed during the middle phase of fruit development. We selected six trees with different O/DM content to investigate *EgGDSL* expression level. The RNA was isolated from fruits harvested at 4 MAF because *EgGDSL* gene had highest expression level in this period. As shown in Fig. 7, there was a positive correlation (*R* = 0.90, *P* = 0.014 < 0.01) between O/DM content and *EgGDSL* gene expression. The expression levels were highest for trees T01 (84.34%) and T02 (82.67%), medium for T03 (78.11%), T04 (76.37%) and T05 (76.17%), and lowest for T06 (72.94%). These results demonstrated that the transcription level of *EgGDSL* gene correlated with the amount of oil content.

## Discussion

The demand for vegetable oil for foods and raw materials has increased considerably in recent years, so the genetic engineering of lipid metabolic pathways and genes involved in triacylglycerol biosynthesis have been studied in oil crops to elevate seed oil content. The lipase and esterase families play important roles in lipid metabolism, regulating the synthesis and hydrolysis of lipids^33^. All enzymes in these families contain a catalytic triad composed of serine, aspartic and histidine residues. Based on their conserved sequences, esterases/lipases can be divided into two families, GXSXG family and GDSL family. GDSL esterase/lipase family usually contains a Gly-Asp-Ser-(Leu) [GDS(L)] motif, with the active serine (Ser) site located closer to the N-terminus^33^. GDSL proteins have been widely found in many plants^33,34^. It was reported that 114 members were revealed from *Oryza sativa*, 53 from *Zea mays*, 108 from *Arabidopsis thaliana* and 96 from *Vitis vinifera*, *et al*.^34,35^. In this study, we aimed to discover novel genes associated with oil content in oil palm. Based on the GWAS data reported by Teh^23^, the p5_sc00003.V1.gene1598 gene which was annotated as GDSL esterase/lipase was selected, because there were 4 significant SNPs (SNP47116, SNP46882, SNP47117 and SNP47120) within a distance of 18 kb from it (Fig. S1). GDSL is an important esterase/lipase family in oil palm as well. A total of 90 genes belonging to the GDSL gene family in oil palm were identified from GenBank. The deduced amino acid sequences of these genes were used to construct the phylogenetic tree. A large protein family commonly contains various domains and repeats that make the member proteins difficult to analyze. The special feature of GDSL esterase/lipase is the presence of the four strictly conserved residues Ser-Gly-Asn-His in conserved blocks I, II, III and V^34,36^. Furthermore, the conserved protein motifs are helpful in improving phylogenetic analysis. Protein motifs were identified using the MEME tool and the top 10 motifs with highest statistical significance (E-value) were selected to evaluate sequence quality. Finally, 71 GDSL genes in oil palm (Table S3) were used to construct the phylogenetic tree after 19 sequences were eliminated. From the results, the motif-based method greatly improved the accuracy of multi-sequences alignment (Fig. 2).

As *EgGDSL* in this research was annotated as GDSL esterase/lipase *At1g71250*-like gene, the *GDSL* homologous genes of *Arabidopsis* should be considered. Ten *GDSL* homologous genes of *Arabidopsis* were used to do phylogenetic and protein motifs analysis together with 71 *GDSL* genes from oil palm. Most GDSL esterase/lipase proteins contain 10 conserved motifs; the presence of the common GDSL motifs affirmed their major functional roles. From Fig. 1, we can see that the 81 *GDSL* genes were divided into five clades. The *EgGDSL* gene was in clade V which included only one other gene (XM_010931430). Furthermore, EgGDSL protein was missing two motifs (motif 2 and 3), perhaps lost during evolution (Fig. 2). Motif 2 is a core sequence of block II in the GDSL family; lack of it may have a certain impact on lipase function. All of these indicate that the *EgGDSL* gene in the present research may have a different function from other *GDSL* genes. The 10 *GDSL* homologous genes of *Arabidopsis* were grouped into clades I and II. Three and five genes had high similarity; they were divided into two subclades, respectively (Fig. 1). Though *EgGDSL* was annotated as GDSL esterase/lipase *At1g71250*-like gene, they appeared to be distant in relationship according to the phylogenetic analysis. Genomic or deduced amino acid sequence alignment also showed a big difference between the *EgGDSL* and *AT1G71250* genes.

We cloned the full-length sequence of the *EgGDSL* gene of oil palm. There were seven SNPs between Dura B and TS3 with 1 SNP in the intron and 6 SNPs in the second exon region; the 6 SNPs in exon 2 caused 4 deduced amino acids differences between DuraB and TS3 (Fig. S3). The predicted structural model of GDSL esterase/lipase protein consists of six α-helices and a central β-sheet core containing six parallel β-strands. The active Ser residue is located in the loop region right after the first β-strands. Block II and III with their Gly and Asn residues are located in the unstructured regions following the second β-sheet and right after the third β, respectively^34^ We analyzed the structure of the deduced EgGDSL protein and found that the three amino acid substitutions (the 93^th^, 163^th^ and 170^th^ amino acids) were in the β-strand, which caused some differences in tertiary protein structures between EgGDSL-5D and EgGDSL-5P (Fig. S6). The positive allele *EgGDSL-5P* was selected for function validation by transformation because the O/B and O/DM content of *Pisifera* were normally much higher than *Dura*. However, the generation of oil palm transformants is difficult because it involves tissue culture and plant regeneration steps. Furthermore, it will take three years for oil palm to bear fruits after planting and about six years to reach full fruit period. Many of the enzymes and genes involved in oil biosynthesis have been proved to be conserved in most of the major oilseed species^37^. Therefore, *Arabidopsis* has been widely used as a model for oilseed crop species^38^, which provides relatively fast and low-cost results before investments in oil palm transformation are made. It was found that expression of *EgGDSL* gene in *Arabidopsis* increased TFA content by 9.5% on average comparing with the wild-type. *EgGDSL* gene had an effect on fatty acid composition as well. The stearic acid (18:0) increased significantly in *GDSL* transgenic lines, whereas the levels of linoleic acid (18:2) and 11-eicosenoic acid were decreased. The results suggest that *EgGDSL* affected oil synthesis. Then we examined the *EgGDSL* expression pattern in oil palm. *EgGDSL* was highly expressed in mesocarp followed by leaf, and the expression level was very low in root. These results were same as those reported^39^. *EgGDSL* expression level also seemed to be consistent with fruit development and oil biosynthesis of oil palm. Oil palm fruits normally complete their development and maturation in 160 days. Oil accumulates in the early phase of fruit development (70 to 100 DAF, days after flowering), and after 120 DAF the amount of oil continues to increase, but at a much lower rate than that in the early stage of development and the fatty acid composition does not change significantly^30^. In our results, the expression level of *EgGDSL* gene began to increase at 2 MAF, reached its peak by 4 MAF, and it declined in the later phase of fruit development (Fig. 6), indicating that *EgGDSL* gene is correlated with oil biosynthesis process.

The GDSL enzyme is a big esterase/lipase family. The remarkably high number of genes in the GDSL family in different plants can be explained by differences in enzyme function and activity on a wide range of substrates^40^. To date, many characteristics of GDSL have not yet been fully and clearly described. *GDSL* genes were found to be associated with morphogenesis, development and disease resistance of the plant^12,41–43^. As an esterase/lipase, *EgGDSL* will be thought firstly to be associated with lipid degradation. Overexpression of *EgGDSL* should lead to decrease in oil content in transgenic lines compared to the wild-type. But we observed opposite results in the present study since expression of *EgGDSL* in *Arabidopsis* led to increase of total fatty acid content, suggesting that *GDSL* is positively correlated with high oil content in oil palm. On the other hand, some studies demonstrating that GDSL lipase was negatively related with oil content were found as well. Huang *et al*.^44^ found that SFAR4, a fatty acid reducer belonging to the GDSL family, reduced the total fatty acid content and changed the composition of unsaturated fatty acids in storage seeds of *Arabidopsis*. Xu *et al*.^45^ identified a lipid-related candidate gene homologous to the GDSL esterase/lipase with function in hydrolase activity. Two GDSL-like lipase genes were significantly downregulated in *myb76* (an MYB transcript factor) mutant of *Arabidopsis* which had increased oil content^46^, indicating that *GDSL* participated in fatty acid catabolism. Therefore, the functions of *GDSL* family were far more complex than we thought. Maybe, *GDSL* affected oil synthesis by regulating hormone metabolism as *Arabidopsis* GDSL lipase 1 (*GLIP1*) has been shown to modulate systemic immunity by regulating ethylene signal^41^. The levels of auxins and cytokinins varied between *DGAT1* transgenic lines and wild-type control^13^. Unfortunately, to date most studies were mainly on the roles of *GDSL* on abiotic stress, pathogen defense and development^34,42,43^. Thus, the mechanisms of *GDSL* genes affecting oil biosynthesis remained to be elucidated. If the transcription factors or proteins interacting with *GDSL* genes were identified, the mechanism of *GDSL* regulating oil biosynthesis would be elucidated.

## Electronic supplementary material

Supplementary information
