# Supplementary material for: Cloning and characterization of EgGDSL, a gene associated with oil content in oil palm
Source: Sci Rep. 2018 Jul 30;8:11406. doi: 10.1038/s41598-018-29492-6 (PMC6065316; doi:10.1038/s41598-018-29492-6)
Supplement: Supplementary file 1 — Supplementary information [file 41598_2018_29492_MOESM1_ESM.pdf]

Supplementary information for the paper

"Cloning and characterization of *EgGDSL*, a gene associated with oil content in oil palm"

Yingjun Zhang[1, 2], Bin Bai[1, 3], May Lee[1], Yuzer Alfiko[4], Antonius Suwanto[4, 5], Gen Hua Yue[1, 6, 7*]

[1] Temasek Life Sciences Laboratory, 1 Research Link, National University of Singapore, Singapore 117604

[2] Institute of Cereal and Oil Crops, Hebei Academy of Agriculture and Forestry Sciences, 162 Hengshan Street, Shijiazhuang, China 050035

[3] Wheat Research Institute, Gansu Academy of Agricultural Sciences, 1 Nongkeyuanxincun, Lanzhou, China 730070

[4] Biotech Lab, Wilmar International, Jakarta, Indonesia

[5] Bogor Agricultural University, Bogor, Indonesia

[6] Department of Biological Sciences, National University of Singapore, Singapore

[7] School of Biological Sciences, Nanyang Technological University, 6 Nanyang Drive, Singapore 637551

* Corresponding author, GH Yue (genhua@tll.org.sg)

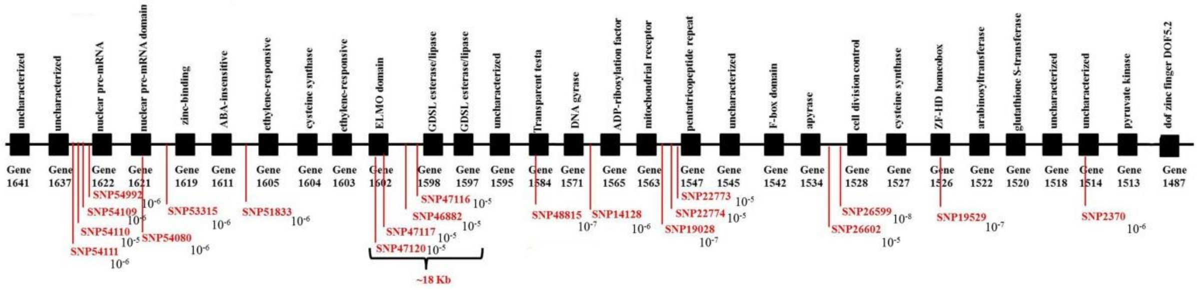

Fig. S1 Sketch map of annotated genes and significant SNPs in scaffold 3 on chromosome 5 of oil palm. The black box indicates gene. Below the box is the number of the gene, and the annotated function of the gene is above the box. The gene number is an abbreviation, for example Gene1598 is a short for p5_sc00003.V1.gene1598. In this diagram, the SNP ID (with *p* value) and location of the SNPs are marked.

```
DuraB      ATGGGACGAAAGCTTCTTCTTCTTGCTCCGGTTTTCGCTCTGGTTTTCTTCGGTAGATTT    60
TS3        ATGGGACGAAAGCTTCTTCTTCTTGCTCCGGTTTTCGCTCTGGTTTTCTTCGGTAGATTT    60

DuraB      TTTCCCAGAGAAATTCCGGACGCCGCAGCGGCGGCGGCCGACGCGGCATATCCGGCGACC   120
TS3        TTTCCCAGAGAAATTCCGGACGCCGCAGCGGCGGCGGCCGACGCGGCATATCCGGCGACC   120

DuraB      GCCCTCTTCGTGCTGGGTGACTCCTCCGTCAACTGTGGCGACAACACCTTCTTCTACCCC   180
TS3        GCCCTCTTCGTGCTGGGTGACTCCTCCGTCAACTGTGGCGACAACACCTTCTTCTACCCC   180

DuraB      CTCCTACCCCTCAACTTCTCCTCCACCTTCTGCAATGGTTCCCACCACCGTCTGCTCCCT   240
TS3        CTCCTACCCCTCAACTTCTCCTCCACCTTCTGCAATGGTTCCCACCACCGTCTGCTCCCT   240

DuraB      GATCTCATCGGTACCCGCGCCTGCTTTAATTTACTTCTTTCTAAGCTTTCTTGATCGGAA   300
TS3        GATCTCATCGGTACCCGCGCCTTCTTTAATTTACTTCTTTCTAAGCTTTCTTGATCGGAA   300

DuraB      ACCCCAAGATTTGATCTCATTGCAAGAAATTAGGGCTTGCTTTCCCCAACAAAAGGGACT   360
TS3        ACCCCAAGATTTGATCTCATTGCAAGAAATTAGGGCTTGCTTTCCCCAACAAAAGGGACT   360

DuraB      GATTCGAAGCTTTCCGATCCGGCTGTTGCAGCCGAGCGGATGGGGCTGCCGCCGGTCACG   420
TS3        GATTCGAAGCTTTCCGATCCGGGTGTTGCAGCCGAGCGGATGGGGCTGCCGCCGGTCGCG   420

DuraB      CCATTCTTCGGCCTGAACGGGACGGCAGCGGCGATGATGCGCGGCGTCAACTTCGGTTCG   480
TS3        CCATTCTTCGGCCTGAACGGGACGGCAGCGGCGATGATGCGCGGCGTCAACTTCGGTTCG   480

DuraB      ACTCCAGCGACGATCGTCTCCGGCGTCCTCGACGGCTGGCTGCGCGGCTACGACCGCTTC   540
TS3        ACTCCAGCGACGATCGTCTCCGGCGTCCTCGACGGCTGGCTGCGCGGCTACGACCGCTTC   540

DuraB      CTCTTCCAGAGCCTCAGCCAGCAGGTCCGCCAGGTGTTCGAGATCCTCCAGGTCCTCCAG   600
TS3        CTCTTCCAGAGCCTCAGCCAGCAGGTCCGCCAGGTGTTCGAGATCCTCCAGGCCCTCCAG   600

DuraB      CTCGAGCTTGGCGCCTCCGCCGCCCGCCGCGCCGCCTCATCCGCCCTCTTCATCCTCTCC   660
TS3        CTCGAGCTCGGCACCTCCGCCGCCCGCCGCGCCGCCTCATCCGCCCTCTTCATCCTCTCC   660

DuraB      TTCGGCAAGGACGACTATGCCGCTCTCTTCTCCCGCGGCTCCGAATCCAACCGCCTCGCC   720
TS3        TTCGGCAAGGACGACTATGCCGCTCTCTTCTCCCGCGGCTCCGAATCCAACCGCCTCGCT   720

DuraB      CCCAAGTACGGACGGCGCGGATTCGCCCGCCTCCTCGTCTCCCGGCTCATCCAGGCCATC   780
TS3        CCCAAGTACGGACGGCGCGGATTCGCCCGCCTCCTCGTCTCCCGGCTCATCCAGGCCATC   780

DuraB      AGGGTAACAACCTATCTCTTGCTCGCTCTTCCATGTGGTGCATCTGATGGATGGCTGTGA   840
TS3        AGGGTAACAACCTATCTCTTGCTCGCTCTTCCATGTGGTGCATCTGATGGATGGCTGTGA   840
```

Fig. S2 Alignment of gene *EgGDSL* of DuraB and TS3 located on oil palm chromosome 5. The open reading frame (ORF) is underlined; the initiation and termination codons are boxed; the SNPs are shadowed.

```
DuraB      MGRKLLLLAPVFALVFFGRFFPREIPDAAAAAADAAYPATALFVLGDSSVNCGDNTFFYP     60
TS3        MGRKLLLLAPVFALVFFGRFFPREIPDAAAAAADAAYPATALFVLGDSSVNCGDNTFFYP     60

DuraB      LLPLNFSSTFCNGSHHRLLPDLIGTDSKLSDPAVAAERMGLPPVTPFFGLNGTAAAMMRG    120
TS3        LLPLNFSSTFCNGSHHRLLPDLIGTDSKLSDPGVAAERMGLPPVAPFFGLNGTAAAMMRG    120

DuraB      VNFGSTPATIVSGVLDGWLRGYDRFLFQSLSQQVRQVFEILQVLQLELGASAARRAASSA    180
TS3        VNFGSTPATIVSGVLDGWLRGYDRFLFQSLSQQVRQVFEILQALQLELGTSAARRAASSA    180

DuraB      LFILSFGKDDYAALFSRGSESNRLAPKYGRRGFARLLVSRLIQAIRVTTYLLLALPCGAS    240
TS3        LFILSFGKDDYAALFSRGSESNRLAPKYGRRGFARLLVSRLIQAIRVTTYLLLALPCGAS    240

DuraB      DGWL                                                            244
TS3        DGWL                                                            244
```

Fig. S3 Alignment of deduced amino acid sequence of gene *EgGDSL* of DuraB and TS3. There are 4 deduced amino acids differences between DuraB and TS3, which are shadowed.

```
DuraB    ATGCCATATCTTCACGGGAGCAACCATTGTAGGTTTTTCCACTCCACGGTCACCCAGAGG    60
TS3      ATGCCATATCTTCACGGGAGCAACCATTGTAGGTTTTTCCACTCCACGGTCACCCAGAGG    60

DuraB    ACTCCCCTGTCTTTATCCCATCCTAATAAACTCCTAGAAGAAGAACGTATTGCCTCTTTG    120
TS3      ACTCCCCTGTCTTTATCCCATCCTAATAAACGCCTAGAAGAAGAACGTATTGCCTCTTTG    120

DuraB    ATCCAAAAATGCCCAGATATGAGAACCCTCCGCCAAATCCACGCTCACTTCCTCAAATTC    180
TS3      ATCCAAAAATGCCCAGATACGAGAACCCTCCGCCAAATCCACGCTCACTTCCTCAAATTC    180

DuraB    CCCGCCAATTCCTCCTCCTTCTACGCCCTCTCCAAGATCCTCGCCTTCTGCGCTCTCTCC    240
TS3      CCCGCCAATTCCTCCTCCTTCTACGCCCTCTCCAAGATCCTCGCCTTCTGCGCTCTCTCC    240

DuraB    CCCTTCGGCGACCTCGCTTACGCCCGCCGCCTGTTCGCCCAAATTCCCCACCCAAATGTC    300
TS3      CCCTTCGGCGACCTCGCTTACGCCCGCCGCCTGTTCGCCCAAATTCCCCACCCAAATGTC    300

DuraB    TTCTCCTGGAACTCCATGATCAGGGGCTCCTCCCAGCTCCCCAACGCCACCAAAGAACCC    360
TS3      TTCTCCTGGAACTCCATGATCAGGGGCTCCTCCCAGCTCCCCAACGCCACCAAAGAACCC    360

DuraB    ATCTTTCTCTACAAACAAATGGTCCAGAAAGGCTTTGCTCTCCCCAATAGCTTCACCCTC    420
TS3      ATCTTTCTCTACAAACAAATGGTCCAGAAAGGCTTTGCTCTCCCCAATAGCTTCACCCTC    420

DuraB    GCCTTCGTTCTTAAAGCTTGCTCGCTTATCTTGGCCTTCTTCGAAGGCAGACAAATCCAT    480
TS3      GCCTTCGTTCTTAAAGCTTGCTCGCTTATCTTGGCCTTCTTCGAAGGCAGACAAATCCAT    480

DuraB    TGCCATGCGTTTAAACACGGGCTTTATTCGAGCCCGTTTGTTCAGACTGGATTGTTGAAT    540
TS3      TGCCATGCGTTTAAACACGGGCTTTATTCGAGCCCGTTTGTTCAGACTGGATTGTTGAAT    540

DuraB    TTTTATGCGAAATGCGAAGAGCTTGTCGCTGCAAGATTGGTTTTTGATGAAATTCCTGAT    600
TS3      TTTTATGCGAAATGCGAAGAGCTTGTCGCTGCAAGATTGGTTTTTGATGAAATTCCTGAT    600

DuraB    AAGAATTTGATCGCTTGGAGCGCGATGATTAGTGGGTACGCGAGGGTGGGGTTGGTGAAT    660
TS3      AAGAATTTGATCGCTTGGAGCGCGATGATTAGTGGGTACGCGAGGGTGGGGTTGGTGAAT    660

DuraB    GAGGCGTTGGAGCTGTTCCGGGAGATGCAGGGGGTGGGGATCAGTCCAGATGAGGTGACA    720
TS3      GAGGCGTTGGAGCTGTTCCGGGAGATGCAGGGGGTGGGGATCAGTCCAGATGAGGTGACA    720

DuraB    ATGGTCAGTGTGATTCAAGCTTGTGCGAAAGCAGGGGCGTTGGATTTAGGGAAGTGGGTG    780
TS3      ATGGTCAGTGTGATTCAAGCTTGTGCGAAAGCAGGGGCGTTGGATTTAGGGAAGTGGGTG    780

DuraB    CACGCTTTTATCGACAGGAATGGAATCAAAGCTGATCTTGAGCTGAAAACTGCGTTAATT    840
TS3      CACGCTTTTATCGACAGGAATGGAATCAAAGCTGATCTTGAGCTGAAAACTGCGTTAATT    840

DuraB    GATATGTATGCCAAGTGTGGGGAGATAGACAGGGCAAGGAAGGTGTTTCATGGGATGGAC    900
TS3      GATATGTATGCCAAGTGTGGGGAGATAGACAGGGCAAGGAAGGTGTTTCATGGGATGGAC    900

DuraB    GTGAGGGATACAAAGGCTTGGAGCTCAATGATTGTTGGCCTGGCGATACATGGACTTGTA    960
TS3      GTGAGGGATACAAAGGCTTGGAGCTCAATGATTGTTGGCCTGGCGATACATGGACTTGTA    960

DuraB    AAAGATGCTTTGGAGCTTTTCTCAAGAATGTTAGAGTCCAAGGTACTGAGATACGGTCTA    1020
TS3      AAAGATGCTTTGGAGCTTTTCTCAAGAATGTTAGAGTCCAAGGTACTGAGATACGGTCTA    1020

DuraB    TGCACAATGTATAGTTATGTGATATGACTTTAGCGTTAAATGGCACAGAAACACATTCTT    1080
TS3      TGCACAATGTATAGTTATGTGATATGACTTTAGCGTTAAATGGCACAGAAACACATTCTT    1080

DuraB    TCCAGCCATGGAGATGAATGAGTGGTGCCTGGAAATAGACTAAAGTCCATAGCTATGTCA    1140
TS3      TCCAGCCATGGAGATGAATGAGTGGTGCCTGGAAATA....AAAGTCCATAGCTATGTCA    1136

DuraB    AAAAATGGATGTACCCACGTTTATGAGTGTCATCCAATATAGAATGACGTTATATCCCTT    1200
TS3      AAAAATGGATGTACCCACGTTTATGAGTGTCATCCAATATAGAATGACGTTATATCCCTT    1196

DuraB    TATTACTGTTCCTAATGTTACAGTATTCTCAATACCATGTAATGCAGCTCCCTAACTTCC    1260
TS3      TATTACTGTTCCTAATGTTACAGTATTCTCAATACCATGTAATGCAGCTCCCTAACTTCC    1256

DuraB    ACTGTTTCATGTACTTTGCAGTAGTTTAAATAATATAATCAGTATATTGCTACTTCTGTT    1320
TS3      ACTGTTTCATGTACTTTGCAGTAGTTTAAATAATATAATCAGTATATTGCTACTTCTGTT    1316
```

```
DuraB      CAGCAATCATAGATACATTACTGCAATCATGGATTTGGTATTTGAATGTATACTGGAAAA   1380
TS3        CAGCAATCATAGATACATTACTGCAATCATGGATTTGGTATTTGAATGTATACTGGAAAA   1376

DuraB      ATTTTGCTTTCAGTTAGAACCAACTGCATGCTTGAAATGAGCATCCTATCTATCTGATAT   1440
TS3        ATTTTGCTTTCAGTTAGAACCAACTGCATGCTTGAAATGAGCATCCTATCTATCTGATAT   1436

DuraB      ACATGATGGATATTACCGATAGTAGTTTCCATCTGTGAAATGTGAATTAATTGTGACACA   1500
TS3        ACATGATGGATATTACCGATAGTAGTTTCCATCTGTGAAATGTGAATTAATTGTGACACA   1496

DuraB      GAATTGGCATATGAAACATTTTTCAATATATTATGTTTTATATGCATGCATGTTTCTGCA   1560
TS3        GAATTGGCATATGAAACATTTTTCAATATATTATGTTTTATATGCATGCATGTTTCTGCA   1556

DuraB      AGCTTGCCAATATGTGGTCTATTGATATGCATGAATTAAAAGTTTCACTTATGAAGTGTA   1620
TS3        AGCTTGCCAATATGTGGTCTATTGATATGCATGAATTAAAAGTTTCACTTATGAAGTGTA   1616

DuraB      CATTTTTCTTGGTGGAACTTTCTTATGACAATTCTTCCATCAATTTTCAGGTGAGGCCTA   1680
TS3        CATTTTTCTTGGTGGAACTTTCTTATGACAATTCTTCCATCAATTTTCAGGTGAGGCCTA   1676

DuraB      ACCATGTGACCTTTATTGGTGTTTTGTCGGCATGTGCTCATAGTGGCTTGGTAAGTGATG   1740
TS3        ACCATGTGACCTTTATTGGTGTTTTGTCGGCATGTGCTCATAGTGGCTTGGTAAGTGATG   1736

DuraB      GTCGGCGATTCTGGTCTATCATGCATGAGTTAGGCATGGAGCCAATGATGGAGCATTATG   1800
TS3        GTCGGCGATTCTGGTCTATCATGCATGAGTTAGGCATGGAGCCAATGATGGAGCATTATG   1796

DuraB      GATGCATGGTTGATCTACTGTGTAGATCTGGCCTTTTTGAAGAAGCTTATTCATTTGTAA   1860
TS3        GATGCATGGTTGATCTACTGTGTAGATCTGGCCTTTTTGAAGAAGCTTATTCATTTGTAA   1856

DuraB      ATACCATGCCTATCATGCCAAATTCCATAATCTGGAGGACCCTTCTGGTAGGATGCAAGA   1920
TS3        ATACCATGCCTATCATGCCAAATTCCATAATCTGGAGGACCCTTCTGGTAGGATGCAAGA   1916

DuraB      GTAATGGATATCTTGATAAAGCAGAGGTTGTTGCAAAGCAACTTCTTGAGTTAGAGCCAC   1980
TS3        GTAATGGATATCTTGATAAAGCAGAGGTTGTTGCAAAGCAACTTCTTGAGTTAGAGCCAC   1976

DuraB      TTAATGCAGAGAATTATGTCCTACTATCTAACCTTTATGCATCCAGCTCTCAGTGGGAAA   2040
TS3        TTAATGCAGAGAATTATGTCCTACTATCTAACCTTTATGCATCCAGCTCTCAGTGGGAAA   2036

DuraB      AGGTGAGCTACATGAGGAAAAAGATGAAGGACAATGGTGTCAAGGTCGTCCCTGGCTGCA   2100
TS3        AGGTGAGCTACATGAGGAAAAAGATGAAGGACAATGGTGTCAAGGTCGTCCCTGGCTGCA   2096

DuraB      GTTCTATTGAAGTTGATGGTTTCTTACGTGAGTTTGTGGTGGGTGATGAGTCGCATCCAG   2160
TS3        GTTCTATTGAAGTTGATGGTTTCTTACATGAGTTTGTGGTGGGTGATGAGTCGCATCCAG   2156

DuraB      AGATCAAGGAGATAAGGAAGGTACTGAGAGAGATAACTGAGAGGGTTCGACTTGTTGGCC   2220
TS3        AGATCAAGGAGATAAGGAAGGTACTGAGAGAGATAACTGAGAGGGTTCGACTTGTTGGCC   2216

DuraB      ATGAGCCATGGACTTCAGCGGTCTTGCATGATGTTGGTGAAGAGGAGAAAGAAGTTGCTC   2280
TS3        ATGAGCCATGGACTTCAGTGGTCTTGCATGATGTTGGTGAAGAGGAGAAAGAAGTTGCTC   2276

DuraB      TGTGCGAGCACAGTGAGAGGTTAGCCATTGCTTTTGGATTGTTGAAAACTAAGGCACCTC   2340
TS3        TGTGCGAGCACAGTGAGAGGTTAGCCATTGCTTTTGGATTGTTGAAAACTAAGGCACCTC   2336

DuraB      TTCCCATTCGGGTGGTAAAGAACTTGAGGGTTTGTAGTGATTGCCATGAGGTGACAAAGA   2400
TS3        TTCCCATTCGGGTGGTAAAGAACTTGAGGGTTTGTAGTGATTGCCATGAGGTGACAAAGA   2396

DuraB      TCATAAGCAAGGCATATGACAGAGAGATTGTTGTAAGGGACCGTGTTCGGTTTCATCGGT   2460
TS3        TCATAAGCAAGGCATATGACAGAGAGATTGTTGTAAGGGACCGTGTTCGGTTTCATCGGT   2456

DuraB      TTGTCAATGGAGCTTGTTCTTGCAATGACTTTTGGTGA                         2498
TS3        TTGTCAATGGAGCTTGTTCTTGCAATGACTTTTGGTGA                         2494
```

Fig. S4 Alignment of gene *EgPPR* of DuraB and TS3 located on oil palm chromosome 5. The open reading frame (ORF) is underlined; the initiation and termination codons are boxed; the SNPs and InDel are shadowed.

```
DuraB      MPYLHGSNHCRFFHSTVTQRTPLSLSHPNKLLEEERIASLIQKCPDMRTLRQIHAHFLKF    60
TS3        MPYLHGSNHCRFFHSTVTQRTPLSLSHPNKrLEEERIASLIQKCPDtRTLRQIHAHFLKF    60

DuraB      PANSSSFYALSKILAFCALSPFGDLAYARRLFAQIPHPNVFSWNSMIRGSSQLPNATKEP   120
TS3        PANSSSFYALSKILAFCALSPFGDLAYARRLFAQIPHPNVFSWNSMIRGSSQLPNATKEP   120

DuraB      IFLYKQMVQKGFALPNSFTLAFVLKACSLILAFFEGRQIHCHAFKHGLYSSPFVQTGLLN   180
TS3        IFLYKQMVQKGFALPNSFTLAFVLKACSLILAFFEGRQIHCHAFKHGLYSSPFVQTGLLN   180

DuraB      FYAKCEELVAARLVFDEIPDKNLIAWSAMISGYARVGLVNEALELFREMQGVGISPDEVT   240
TS3        FYAKCEELVAARLVFDEIPDKNLIAWSAMISGYARVGLVNEALELFREMQGVGISPDEVT   240

DuraB      MVSVIQACAKAGALDLGKWVHAFIDRNGIKADLELKTALIDMYAKCGEIDRARKVFHGMD   300
TS3        MVSVIQACAKAGALDLGKWVHAFIDRNGIKADLELKTALIDMYAKCGEIDRARKVFHGMD   300

DuraB      VRDTKAWSSMIVGLAIHGLVKDALELFSRMLESKVRPNHVTFIGVLSACAHSGLVSDGRR   360
TS3        VRDTKAWSSMIVGLAIHGLVKDALELFSRMLESKVRPNHVTFIGVLSACAHSGLVSDGRR   360

DuraB      FWSIMHELGMEPMMEHYGCMVDLLCRSGLFEEAYSFVNTMPIMPNSIIWRTLLVGCKSNG   420
TS3        FWSIMHELGMEPMMEHYGCMVDLLCRSGLFEEAYSFVNTMPIMPNSIIWRTLLVGCKSNG   420

DuraB      YLDKAEVVAKQLLELEPLNAENYVLLSNLYASSSQWEKVSYMRKKMKDNGVKVVPGCSSI   480
TS3        YLDKAEVVAKQLLELEPLNAENYVLLSNLYASSSQWEKVSYMRKKMKDNGVKVVPGCSSI   480

DuraB      EVDGFLREFVVGDESHPEIKEIRKVLREITERVRLVGHEPWTSAVLHDVGEEEKEVALCE   540
TS3        EVDGFLhEFVVGDESHPEIKEIRKVLREITERVRLVGHEPWTSvVLHDVGEEEKEVALCE   540

DuraB      HSERLAIAFGLLKTKAPLPIRVVKNLRVCSDCHEVTKIISKAYDREIVVRDRVRFHRFVN   600
TS3        HSERLAIAFGLLKTKAPLPIRVVKNLRVCSDCHEVTKIISKAYDREIVVRDRVRFHRFVN   600

DuraB      GACSCNDFW                                                      609
TS3        GACSCNDFW                                                      609
```

Fig. S5 Alignment of deduced amino acid sequence of gene *EgPPR* of DuraB and TS3. There are 4 deduced amino acids differences between DuraB and TS3, which are shadowed.

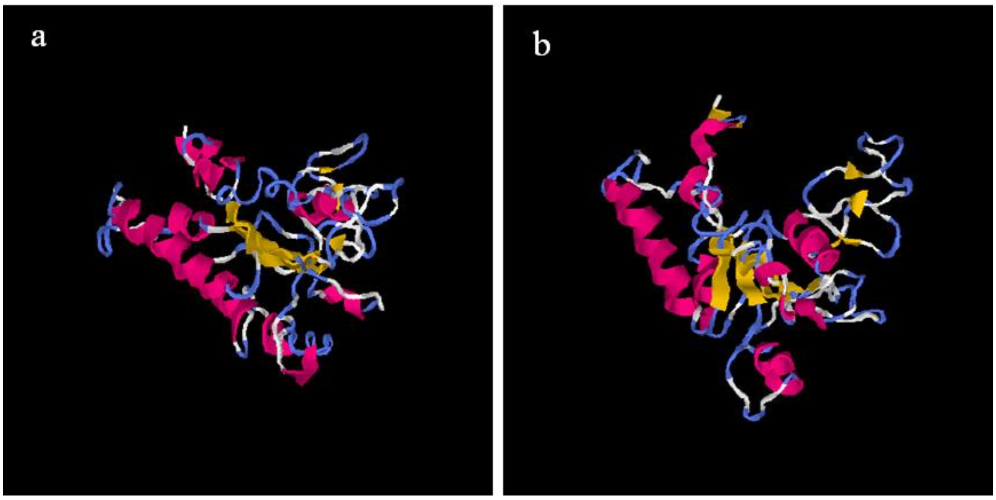

Fig. S6 Tertiary protein structures of EgGDSL-5D and EgGDSL-5P. (a) EgGDSL-5D; (b) EgGDSL-5P.

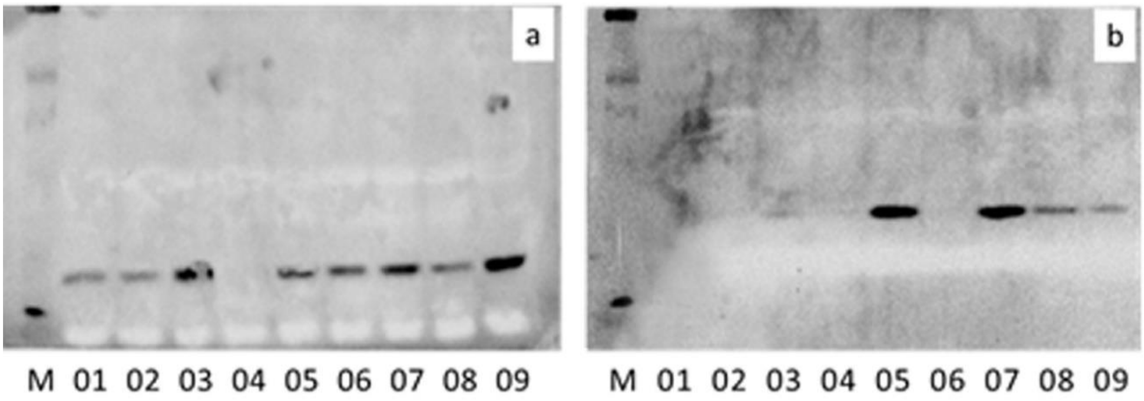

Fig. S7 Western blot analysis of EgGDSL and EgPPR in different transformation lines. (a) EgGDSL; (b) EgPPR. M indicates the protein marker, and the number 01 to 09 means different transformation lines. It is to note that the picture of the gels (a and b) were cut from two different gels, and the loading wells were not included in the pictures during taking photos. The exposure time of the two gels might be different but does not affect the interpretation of the results.

Table S1 The list of trees selected for *EgGDSL* gene expression level analysis

Tree No.	Oil to bunch (O/B)				Oil to dry mesocarp (O/DM)		
	2012	2013	2014	Average	2012	2013	Average
T01	33.78	33.20	33.94	33.64	83.37	85.31	84.34
T02	30.70	34.07	34.40	33.06	83.67	81.67	82.67
T03	34.24	29.37	33.70	32.44	78.46	77.76	78.11
T04	24.20	23.15	24.44	23.93	75.48	77.26	76.37
T05	25.31	21.83	27.42	24.85	78.49	73.85	76.17
T06	24.40	24.00	20.54	22.98	71.03	74.85	72.94

Table S2 The list of SNPs with $p < 10^{-5}$ on chromosome 5 of oil palm

No.	SNP ID	Physical position	*p* value	Scaffold
1	SNP20192	40,916,454	7.76E-05	sc_3
2	SNP20190	40,902,353	5.38E-05	
3	SNP02370	40,396,733	9.48E-06	
4	SNP19529	40,300,709	1.51E-07	
5	SNP26599	40,269,392	1.66E-08	
6	SNP26602	40,249,343	9.19E-05	
7	SNP22773	40,129,189	5.13E-05	
8	SNP22774	40,112,108	8.60E-05	
9	SNP19028	40,066,844	2.35E-07	
10	SNP14128	39,966,907	7.07E-06	
11	SNP48815	39,860,983	9.14E-07	
12	SNP47116	39,806,797	3.07E-05	
13	SNP46882	39,805,514	2.97E-05	
14	SNP47117	39,804,720	3.97E-05	
15	SNP47120	39,799,450	2.99E-05	
16	SNP51833	39,763,460	1.77E-06	
17	SNP53315	39,653,455	1.58E-06	
18	SNP54080	39,642,505	6.16E-06	
19	SNP54992	39,620,126	4.17E-06	
20	SNP54109	39,613,906	4.17E-06	
21	SNP54110	39,610,847	1.56E-05	
22	SNP54111	39,607,208	6.15E-06	
23	SNP24845	39,210,662	3.89E-09	
24	SNP30409	36,234,729	1.52E-05	sc_86
25	SNP30440	36,218,554	2.07E-05	
26	SNP33957	36,158,880	1.10E-05	
27	SNP24664	35,121,695	2.29E-05	sc_94
28	SNP24668	35,100,527	4.02E-07	
29	SNP48207	35,080,572	3.51E-05	
30	SNP42931	34,980,654	6.07E-06	
31	SNP15219	34,863,191	2.28E-05	

32	SNP15218	34,856,258	1.02E-08
33	SNP10418	34,828,628	2.39E-06
34	SNP22444	34,773,282	2.11E-05
35	SNP40073	34,612,126	3.20E-07
36	SNP37422	34,603,394	7.09E-06
37	SNP18372	34,525,454	9.70E-07
38	SNP18373	34,521,370	3.34E-05
39	SNP03832	34,454,937	4.68E-05
40	SNP16161	33,975,929	1.64E-05
41	SNP39298	33,457,617	7.84E-05
42	SNP49049	32,866,989	4.22E-08

Table S3 The list of GDSL genes selected for phylogenetic and protein motifs analysis

Gene No.	Accession number /Locus tag	Organism	Gene No.	Accession number /Locus tag	Organism
1	KY911278	Oil palm	42	XM_010923142	Oil palm
2	XM_010906932	Oil palm	43	XM_010923268	Oil palm
3	XM_010908624	Oil palm	44	XM_010941499	Oil palm
4	XM_010909474	Oil palm	45	XM_010924708	Oil palm
5	XM_010909570	Oil palm	46	XM_010940491	Oil palm
6	XM_010909738	Oil palm	47	XM_010924979	Oil palm
7	XM_010911693	Oil palm	48	XM_010940408	Oil palm
8	XM_010911695	Oil palm	49	XM_010937779	Oil palm
9	XM_010912814	Oil palm	50	XM_010926389	Oil palm
10	XM_010913333	Oil palm	51	XM_010936910	Oil palm
11	XM_019850584	Oil palm	52	XM_010927106	Oil palm
12	XM_010914283	Oil palm	53	XM_010927390	Oil palm
13	XM_019849876	Oil palm	54	XM_010927391	Oil palm
14	XM_010914330	Oil palm	55	XM_010927394	Oil palm
15	XM_010914357	Oil palm	56	XM_010927485	Oil palm
16	XM_010914369	Oil palm	57	XM_010936909	Oil palm
17	XM_010914575	Oil palm	58	XM_010930101	Oil palm
18	XM_010914734	Oil palm	59	XM_010931336	Oil palm
19	XM_010915086	Oil palm	60	XM_010931430	Oil palm
20	XM_010915100	Oil palm	61	XM_010931431	Oil palm
21	XM_010916374	Oil palm	62	XM_010932057	Oil palm
22	XM_010916375	Oil palm	63	XM_010932121	Oil palm
23	XM_010917046	Oil palm	64	XM_010932248	Oil palm
24	XM_019846306	Oil palm	65	XM_010935054	Oil palm
25	XM_010917304	Oil palm	66	XM_010935097	Oil palm
26	XM_010917305	Oil palm	67	XM_010935800	Oil palm
27	XM_010917306	Oil palm	68	XM_010936828	Oil palm
28	XM_010917307	Oil palm	69	XM_010936830	Oil palm
29	XM_010917308	Oil palm	70	XM_010936906	Oil palm
30	XM_019846300	Oil palm	71	XM_010936908	Oil palm
31	XM_010917742	Oil palm	72	AT5G45950	Arabidopsis
32	XM_010919169	Oil palm	73	AT5G40990	Arabidopsis
33	XM_010944978	Oil palm	74	AT1G71250	Arabidopsis
34	XM_010944925	Oil palm	75	AT5G08460	Arabidopsis
35	XM_010943196	Oil palm	76	AT2G03980	Arabidopsis
36	XM_010942383	Oil palm	77	AT5G15720	Arabidopsis
37	XM_010920230	Oil palm	78	AT3G14225	Arabidopsis
38	XM_010920633	Oil palm	79	AT1G53940	Arabidopsis

39	XM_010921117	Oil palm	80	AT1G53920	Arabidopsis
40	XM_010921804	Oil palm	81	AT1G53990	Arabidopsis
41	XM_010921805	Oil palm			
